# ADEVO: Proof-of-concept of adenovirus-directed EVOlution by random peptide display on the fiber knob

**DOI:** 10.1016/j.omton.2024.200867

**Published:** 2024-08-30

**Authors:** Erwan Sallard, Julian Fischer, Katrin Schroeer, Lisa-Marie Dawson, Nissai Beaude, Arsalene Affes, Eric Ehrke-Schulz, Wenli Zhang, Adrian Westhaus, Marti Cabanes-Creus, Leszek Lisowski, Zsolt Ruszics, Anja Ehrhardt

**Affiliations:** 1 Virology and Microbiology, Centre for Biomedical Education & Research (ZBAF), Faculty of Health, Witten/Herdecke University, 58453 Witten, Germany; 2 Institute of Virology, Faculty of Medicine, University Medical Center Freiburg, University of Freiburg, Freiburg, Germany; 3 AgroParisTech, Paris-Saclay University, Palaiseau, France; 4 Translational Vectorology Research Unit, Children’s Medical Research Institute, Faculty of Medicine and Health, The University of Sydney, Westmead, NSW 2145, Australia; 5 Australian Genome Therapeutics Centre, Children’s Medical Research Institute and Sydney Children’s Hospitals Network, Westmead, NSW 2145, Australia; 6 Military Institute of Medicine – National Research Institute, Laboratory of Molecular Oncology and Innovative Therapies, 04-141 Warsaw, Poland

**Keywords:** MT: Regular Issue, adenovirus vector, vector library, directed evolution, vector retargeting, oncolytic virotherapy

## Abstract

Directed evolution of viral vectors involves the generation of randomized libraries followed by artificial selection of improved variants. Directed evolution only yielded limited results in adenovirus (AdV) engineering until now, mainly due to insufficient complexities of randomized libraries. Meanwhile, clinical applications of AdVs as gene therapy or oncolytic vectors are still hampered by the predetermined tropism of natural types. To overcome this challenge, we hypothesized that randomized peptide insertions on the capsid surface can be incorporated into the AdV bioengineering toolbox for retargeting. Here we developed AdV-directed EVOlution protocols based on fiber knob peptide display. Human AdV-C5-derived libraries were constructed following three distinct protocols and selected on a panel of cancer cell lines, with the goal of identifying variants able to infect and lyse these tumor cells more efficiently. All protocols enabled the construction of high complexity libraries with up to 9.6 × 10^5^ unique variants, an approximate 100-fold improvement compared with previously published AdV libraries. After selection, the most enriched variants, which were robustly selected in various cancer cell lines, did not display enhanced infectivity but rather more efficient replication and cell lysis. Selected inserts also conferred enhanced lysis ability to oncolytic AdVs restricted to telomerase-expressing cell lines.

## Graphical abstract

**Figure:**
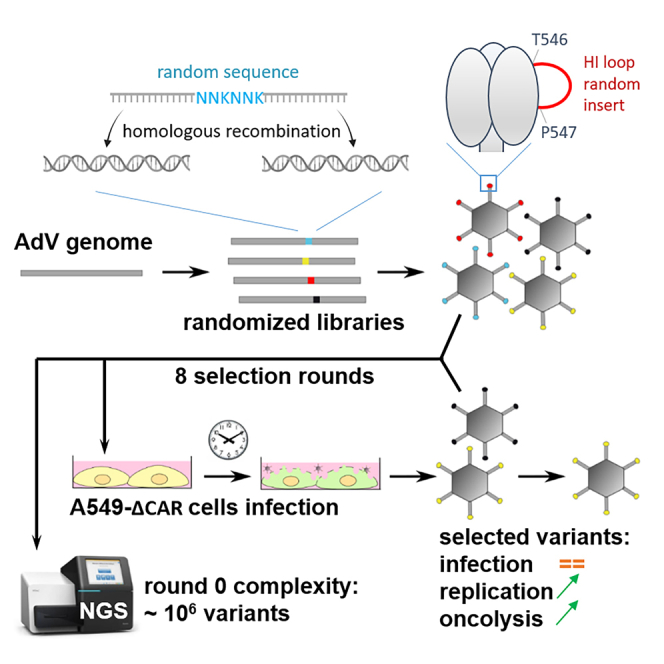

## Teaser

Directed evolution can facilitate the generation and selection of viral vectors with improved features for clinical applications. Here, Sallard and colleagues describe protocols for AdV high-diversity library generation and apply them to select broad-spectrum highly lytic variants for oncolytic virotherapy approaches.

## Introduction

Adenoviruses (AdVs) are double-stranded DNA viruses with linear genomes of 26–45 kb and usually cause asymptomatic or benign respiratory or digestive infections in humans.^1^ Several AdV types, most notably human AdV type 5 (HAdV-C5) from species C, have been genetically engineered to construct adenoviral vectors for vaccines, gene therapy, or oncolytic virotherapy. Due to their ability to selectively replicate in and lyse cancer cells, deliver transgenes, and locally recruit the immune system, oncolytic AdVs (OAdVs) can complement already approved therapies such as chemotherapies and immunotherapies. Dozens of OAdVs are currently undergoing clinical trials^2^ and the OAdV Oncorine (H101) was approved by the Chinese Drug Agency for the treatment of nasopharyngeal carcinoma as early as 2005.^3^ However, no other OAdV has been approved for clinical use to date.

The knob domain of the fiber protein is responsible for the binding of natural AdV types to their cellular primary receptors and thereby contributes to tropism determination. There are few AdV primary receptors and HAdV-C5 predominantly uses the coxsackievirus and AdV receptor (CAR).^4^ AdV receptors are expressed in numerous cell types, both healthy and tumorous, at varying levels.^5^ For example, CAR expression levels are high on erythrocytes and in the juvenile brain but very low in several cancer types, including pancreatic^6^ and colon adenocarcinomas, as well as prostate cancer.^6^^,^^7^ Therefore, most potential HAdV-C5-based OAdVs would transduce these tumor types inefficiently while displaying high levels of off-target transduction of healthy tissues, prompting safety issues and further decreasing the number of virus particles (VPs) able to transduce the target tissues. This is particularly concerning given that the cited tumor types still have a very high lethality and medicine would greatly benefit from efficient OAdVs directed against them. Overall, the lack of tropism specificity and the low transduction efficiency are two of the main hindrances in AdV clinical applications, even in case of local delivery.^5^ Therefore, retargeted AdVs are in high demand.

Several approaches to AdV capsid genetic or chemical engineering have been harnessed to increase the efficiency or specificity of transduction in target tissues. In instances where a target receptor as well as its ligands were known, rational design studies showed that the insertion of short peptides, for example, octopeptides, in exposed loops of the fiber knob domain such as the HI loop can lead to successful AdV retargeting.^8^^,^^9^^,^^10^ However, rational design suffers from low throughput and is not applicable to numerous target cell types for which the optimal vector modifications are not known in advance. This warrants the establishment of new AdV engineering workflows based on complementary principles.

**Figure 1:**
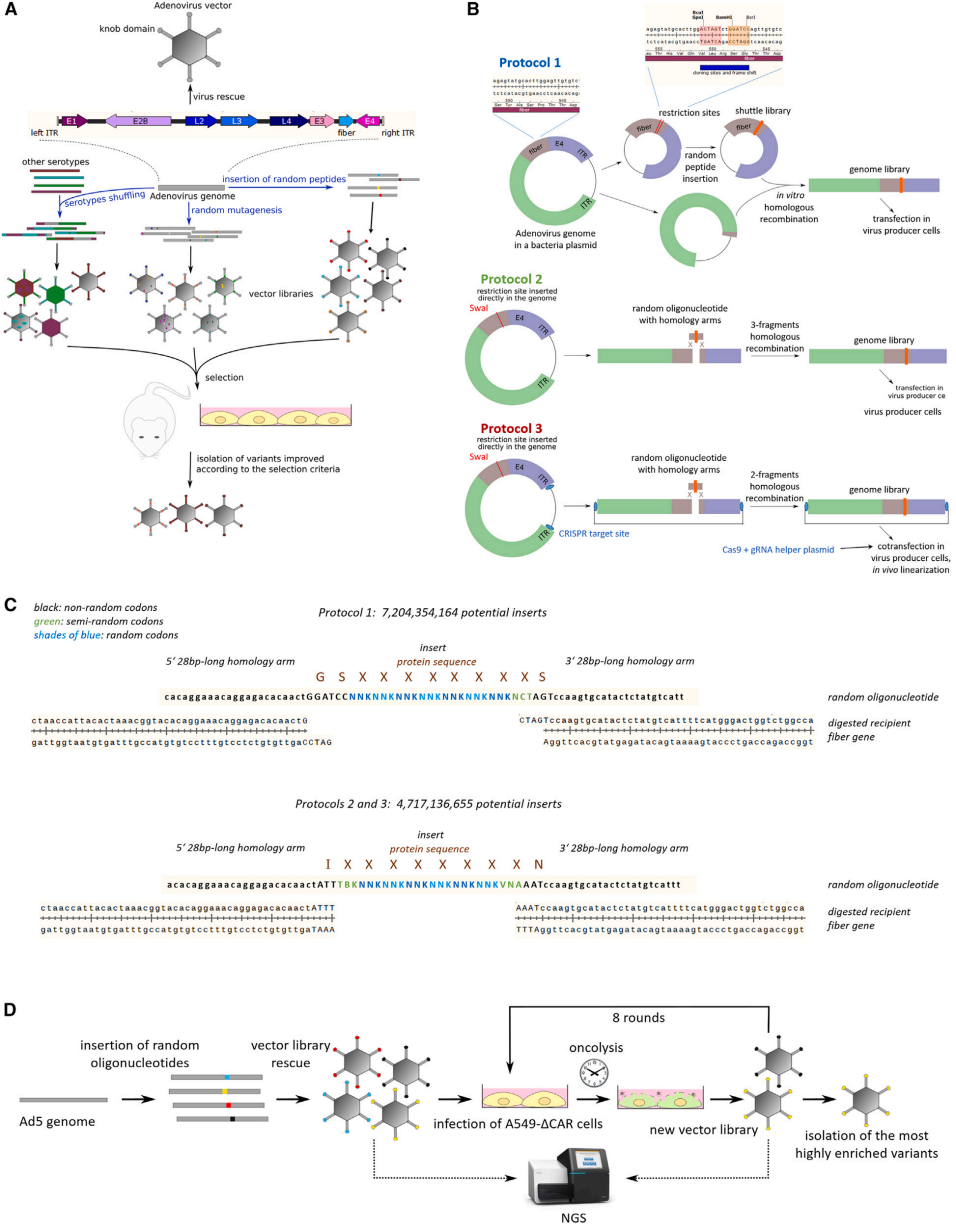
Directed evolution workflows (A) Directed evolution principles. Random libraries can be generated by shuffling of pre-existing viruses, random mutagenesis, or random peptide display. Libraries are subsequently selected in chosen models under stringent conditions to enrich variants optimizing the desired criteria. The schematic of AdV genome represents the ITRs and most major genes at a scale representative of HAdV-C5 WT genome. (B) ADEVO protocols for library generation studied in this article. All protocols are based on the insertion of oligonucleotides containing eight random or semi-random codons in the HI loop of the fiber knob domain. In protocol 1, libraries are first built in a fiber-carrying shuttle plasmid. The shuttle plasmid is then recombined with a truncated AdV genome to reassemble full insert-carrying AdV genomes. In protocol 2, libraries are built in a single step by three-fragment homologous recombination between the left and right parts of the AdV genome and the random oligonucleotide. Protocol 3 involves a single-step, two-fragment homologous recombination, and an *in vivo* Cas9-mediat`ed AdV genome linearization after transfection. (C) Sequence details of peptide insertions. Random oligonucleotides used for homologous recombination inside the fiber gene HI loop contain 28-nt-long homology arms matching the fiber (lowercase) and restriction sites (uppercase) sequences of the different protocols, and a central insert of 8 random or semi-random codons. (D) Proof-of-concept experiment design. Several libraries were constructed and submitted to eight rounds of selection on A549-ΔCAR cells. Full results are available for one protocol 1 library, two protocol 2 libraries and one protocol 3 library. Library quality was assessed by NGS at different steps of the directed evolution workflow. After selection, the most highly enriched variants were isolated and characterized.

Directed evolution, a concept inspired by the natural processes of random mutation followed by positive selection of the fittest, holds the potential to overcome these shortcomings and substantially accelerate vector bioengineering due to its high throughput and unbiased variant testing. Viral vector directed evolution involves the production of randomized libraries containing thousands of vector variants followed by the selection of the most efficient variants for a given purpose. Random libraries can be produced by (1) insertion of random peptides at specific sites in viral proteins; (2) shuffling of different pre-existing viruses to produce chimeric vectors; and/or (3) random mutagenesis of pre-existing vectors (Figure 1A).^11^ The second approach was successfully used with OAdVs in 2008 to obtain ColoAd1, also known as EnOncoAd.^12^ ColoAd1 is an Ad3-Ad11 chimera produced by recombination of species B AdV genomes and selected by serial passage of the replication-competent variant library on colon adenocarcinoma cell lines. This OAdV (under the name Enadenotucirev) and its derivatives have been used in several clinical trials for oncolytic virotherapy.^13^ However, it remains to this day the only example of successful directed evolution AdV bioengineering. Furthermore, random peptide display was used extensively with certain vector systems such as adeno-associated viruses (AAVs), yielding for example the AAV2/7m8 vector^14^ that is now being investigated in several clinical trials of retina gene therapy (NCT03748784 and NCT03748784), but despite this success random peptide display was scarcely tested in AdVs. The rarity of AdV-directed evolution (ADEVO) studies can be attributed to the technical difficulties of cloning libraries of these large viruses, limiting the variability and size of variant libraries and thus the potency of directed evolution. Indeed, the library complexities (i.e., the number of unique variants present in a library) reported in previous studies of AdV random peptide display reached at best an order of magnitude of only ten thousands,^15^ while complexities of 5 million or more have already been reported with AAVs.^16^ Although these small AdV libraries facilitated the selection of replication-competent AdVs (RC-Ads) with improved cancer cell lysis notably in the AsPc1 pancreatic adenocarcinoma cell line *in vitro*^17^ as well as *in vivo* in peritoneal tumor xenografts,^18^ to our knowledge the selected variants did not make it to clinical trials. Nevertheless, novel high-efficiency genome assembly tools based on Gibson Assembly derivatives may facilitate higher throughput AdV library cloning and were already successfully used in AdV rational design applications.^19^

Here, we designed and optimized new protocols for AdV library generation and assessed them in a proof-of-concept experiment with the goal of selecting improved HAdV-C5-derived RC-Ads targeting a CAR-deleted A549 cell line as a model for oncolysis applications. RC-Ad libraries were built and selected by serial passaging on target cells, and library complexity and composition was followed by deep sequencing during selection. We show that all three workflows tested facilitated the generation of highly complex libraries comprising hundreds of thousands of unique variants, and the selection of RC-Ads with increased lytic effect in the model cell line. Subsequently, selections were conducted in additional cancer cell lines, and identified lytic fiber variants were incorporated in replication-selective AdVs.

## Results

### ADEVO protocols facilitate the production of highly complex random libraries

We designed three protocols for ADEVO. Protocol 1 was inspired by AAV random library generation methods^20^: HAdV-C5 fiber, E4 region, and right inverted terminal repeat (ITR) were subcloned into a 7-kb-long shuttle plasmid, corresponding with the size of typical AAV plasmids. BamHI and SpeI restriction sites were inserted into the fiber HI loop region (Figure 1B) so that random oligonucleotides containing seven random codons and one semi-random codon could be inserted into the shuttle plasmid by high-throughput homologous recombination (Figure 1C). Obtained shuttle library plasmids were linearized and recombined with plasmids carrying all HAdV-C5 sequences absent from the shuttle plasmid, namely, those from the left ITR to the U exon, to reassemble functional HAdV-C5-derived genomes carrying random inserts in their fiber gene. Reassembled genome libraries were transfected into producer cells to rescue the round-0 VP libraries. To minimize the risk of library contamination by insert-less religated AdV genomes, two nucleotides were inserted in the shuttle plasmid between the BamHI and SpeI restriction sites so that insert-less shuttle plasmids would give rise after genome reassembly to non-functional genomes with a frameshift in the fiber gene.

Alternatively, ADEVO protocol 2 consists of a single-step library generation whereby a SwaI restriction site, absent from the HAdV-C5-wild type (WT) genome, was inserted at the chosen position in the fiber HI loop region (Figure 1B). After digestion of the assembly backbone, three-fragment homologous recombination was conducted between a random oligonucleotide and the two backbone genome fragments left and right of the SwaI site (Figure 1C). Here, random oligonucleotides contained six random and two semi-random codons to minimize differences in the maximal theoretical library complexity between both protocols since the flanking sequences were different from protocol 1. Like in protocol 1, reassembled protocol 2 genome libraries can be transfected into producer cells to rescue round-0 VP libraries, then undergo selection. Likewise, the SwaI restriction site introduced a frameshift and a stop codon, which ensured that parental assembly backbones would not contaminate the VP libraries.

AdV rescue was reported to be less efficient after transfection of already linearized AdV genomes than after transfection of circular plasmids carrying AdV genomes followed by in-cell genome linearization by CRISPR-Cas9.^21^ We thus developed protocol 3 derived from protocol 2 to use this improved transfection method. To do so, we replaced the SwaI and PacI restriction sites in the bacterial backbone of the parental AdV genome plasmid with CRISPR-Cas9 target sites so that the genome reassembly products remain circular and can be linearized in producer cells after co-transfection with helper plasmids expressing Cas9 and the specific guide RNA (gRNA) (Figure 1B).

Each protocol was optimized with the goals of maximizing purity and yield at each step, and of minimizing cross-packaging. Cross-packaging occurs when a capsid carries a genome encoding for a different capsid sequence.^22^ In directed evolution experiments, this phenotype-genotype discrepancy can cause selection failure, as selected capsid properties are lost at the next virus generation. We quantified for the first time AdV cross-packaging, in the context of virus rescue after AdV genome transfection, corresponding with library production setting. We detected dose-dependent rates of cross-packaging and found that transfection of 5,000 AdV genomes per cell or less facilitated high titer virus rescue while keeping cross-packaging rates below 3% (Figure S1). We chose to use these conditions for library generation.

Next, we designed a proof-of-concept experiment with the aim of comparing the ADEVO protocols and establishing the feasibility and usefulness of directed evolution for AdVs. Since improved oncolytic vectors are in high demand for lung cancer,^23^ we used A549-ΔCAR cells, derived from the A549 lung adenocarcinoma model cell line by knock-out of the primary receptor of WT HAdV-C5^24^ as target cells for our libraries. It could be expected that highly complex libraries of fiber-modified HAdV-C5 variants would contain AdVs able to efficiently infect lung cancer cells through CAR-independent mechanisms. To select for such variants retargeted to A549-ΔCAR cells, cells were infected with RC-Ad libraries and, after replication, progeny RC-Ads were harvested as a new library for subsequent selection rounds. Overall, eight rounds of iterative selection were performed per ADEVO library, and the final enriched variants were identified by next-generation sequencing (NGS) (Figure 1D). Finally, the efficiency of ADEVO protocols was assessed depending on round-0 library purity and complexity, and the cancer cell lysis ability of the selected variants in A549-ΔCAR cells.

To estimate the total library complexity at round 0 (which is unlikely to be fully captured by deep sequencing of a single aliquot), we assessed several statistical models of capture-recapture (Figure S2A). These models were developed with the aim of estimating animal populations by capturing, marking, and releasing animals, then capturing a new population aliquot and counting how many of the animals had already been identified in the first sampling. Here we adapted these models by sequencing several library aliquots, considering each insert peptide sequence as an individual animal and counting in how many aliquots it was identified. We used so-called Mh-type models, which take into account that variants have different probabilities of being sampled in none, one or several aliquots due to abundance differences; and Mth-type models, which in addition account for varying aliquot sizes. We found Anne Chao’s estimator^25^ to be the most consistent for our data and used it henceforth. Of note, it is a conservative estimator designed to indicate the lower bound of the confidence interval of population size.

**Figure 2:**
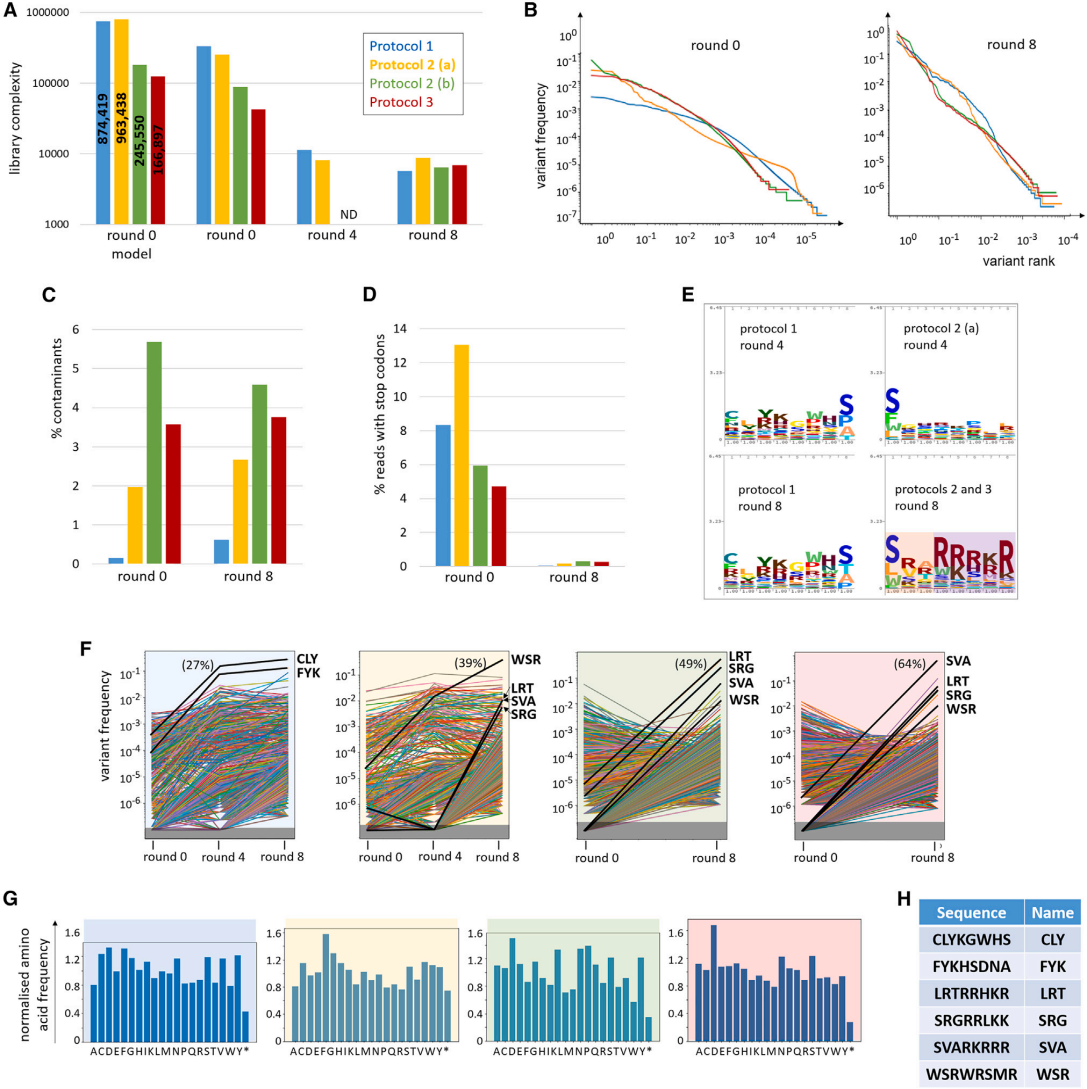
Libraries complexity, purity and composition before and after selection One library assembled following protocol 1, two following protocol 2 (termed “a” and “b”) and one following protocol 3 underwent eight selection rounds on A549-ΔCAR cells. All libraries were sequenced by NGS after rounds 0 and 8, as well as libraries 1 and 2(a) after round 4. (A) Statistical modeling estimation of total round 0 library complexity (“round 0 model”), or number of variants identified by NGS (“round 0,” “round 4,” “round 8”). (B) Proportion of round 0 (left) and round 8 (right) libraries occupied by each variant, ranked in decreasing abundance. (C) Contaminants (parental genomes, inserts with incorrect length, variants with mutations outside the insert, etc) identified by NGS remained rare in the libraries. (D) Percentage of all NGS reads containing stop codons in the insert sequence. The loss of inserts with stop codons across selection rounds indicates that non-functional variants were counter-selected. (E) Logo profiles of insert sequences in selected libraries. The amino acid sequences of the insert random and semi-random positions of the 100 most frequent variants of the considered library were aligned, with a number of repeats per variant proportional to their detected frequency in the library. For round 8, the protocol 2 and 3 libraries were combined given that they have the same insert flanking sequences (SwaI restriction site) and the most highly enriched variants of these libraries largely overlapped. After eight rounds of selection, a distinct pattern emerges in protocol 2 and 3 libraries: mostly hydrophobic residues in the first three positions, strong predominance of positively charged residues in the last five. (F) Evolution of variant frequencies within libraries during selection. The most highly enriched variants (bold lines) were chosen for purification. In brackets: proportion of the library occupied by the dominant variant after selection. Black rectangle, undetected variants. Only variants detected at round 8 are represented. (G) Frequencies of each amino acid at selection round 0 within the fully random insert sequence normalized on theoretical expected frequencies show limited initial sequence bias. (H) Most highly enriched variants in the different libraries at the end of eight selection rounds. They will be referred to in the text by the name indicated in the right column. (H) Dominant variants chosen for purification.

A total of four libraries underwent the entire directed evolution process: one from protocol 1, two from protocol 2, and one from protocol 3. Their estimated complexity at round 0 reached more than 963,000 for one protocol 2 library and 245,000 for the other, while the protocol 1 library counted more than 874,000 unique variants estimated and the protocol 3 library more than 166,000 (Figure 2A). The observed complexity in the sequenced library aliquots was, respectively, more than 253,000, 88,000, 334,000, and 42,000 (Figure S2B). Protocols 2 and 3 round 0 libraries showed already large differences in individual variants relative abundance (Figure 2B), while the protocol 1 library variants abundance distribution was flatter. The library purity ranged between 94% and 98% for protocols 2 and 3 libraries while reaching well over 99% for the protocol 1 library (Figure 2C).

### Selection of improved RC-AdVs and OAdVs

All four libraries were submitted to eight rounds of selection each on A549-ΔCAR cells. As anticipated, library complexity decreased (Figure 2A). Variants with stop codons in their inserts were virtually eliminated from the libraries (Figure 2D), showing that non-functional variants had been counter-selected. Meanwhile, library purity remained approximately constant across selection rounds (Figure 2C). The dominant variant of each library reached 27%–64% abundance within the entire library (Figures 2B and 2F). The abundance distribution had become substantially steeper than at round 0. A limited number of variants had been highly enriched; several hundreds of variants subsisted at low frequency, representing presumably bystander variants that had been able to infect and replicate in the target cells but at a disadvantage compared with the enriched variants; finally, thousands of variants had been eliminated from the libraries during selection (Figure 2B).

A distinctive insert sequence pattern could be observed in protocols 2 and 3 libraries at selection round 8, with the first three semi-random and random positions predominantly occupied by hydrophobic amino acids, contrasting with the strong enrichment of positively charged amino acids in the last five positions (Figure 2E). This pattern was not clearly detectable at round 4 (Figure 2E), and the amino acid distribution at round 0 showed little enrichment or reduction in any amino acid (Figure 2G), indicating that the pattern was not the result of initial biases in library construction. The protocol 1 library did not display distinctive insert sequence motifs, which may be attributed to the different insert flanking sequences compared with protocols 2 and 3.

The most highly enriched variants in the different libraries were identified (Figure 2H), re-cloned, and purified. Interestingly, the sets of most highly enriched variants from protocols 2 and 3 libraries largely overlapped (Figure 2F).

**Figure 3:**
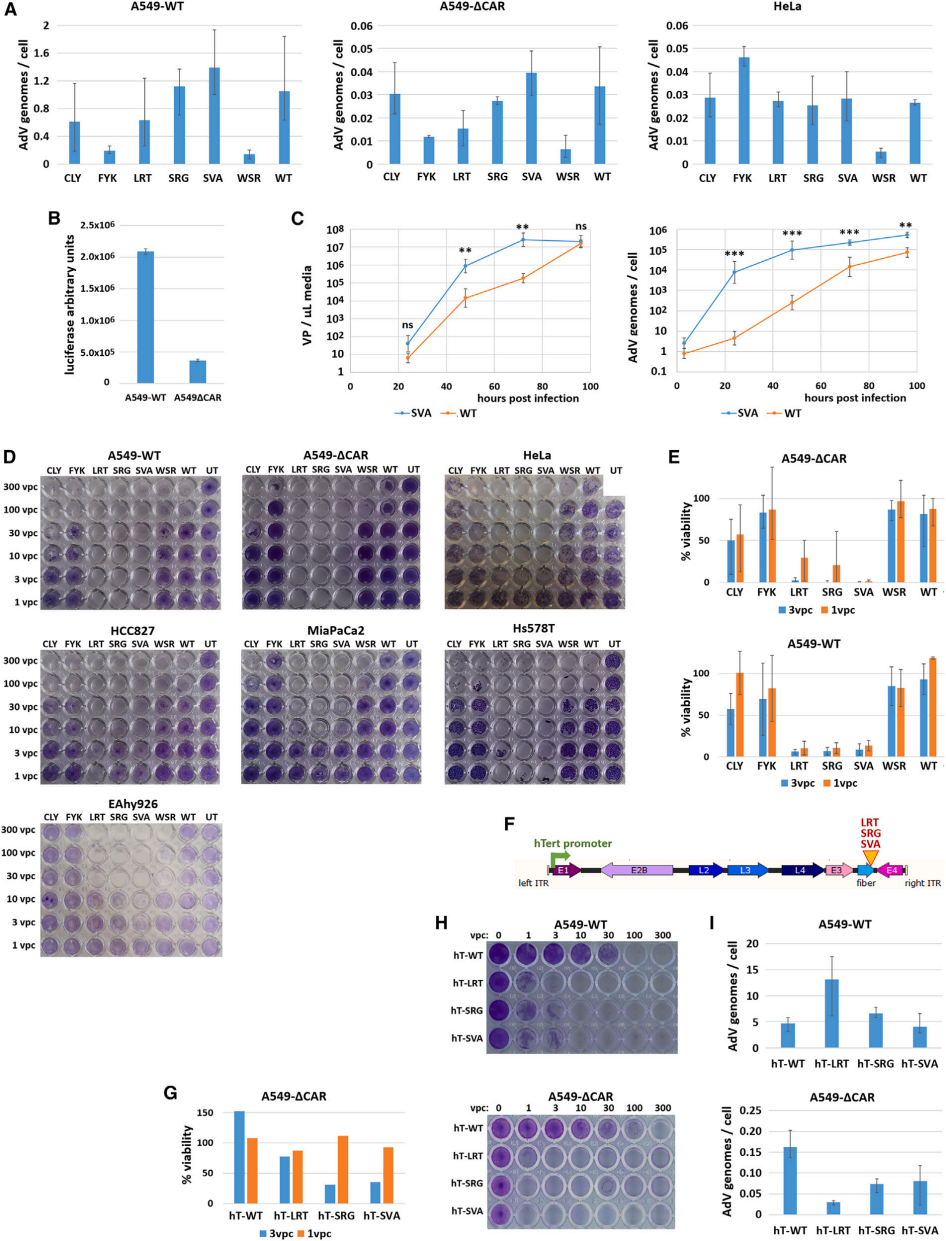
ADEVO-selected variants show improved cell lysis and DNA replication but not increased infectivity (A) ADEVO-selected variants do not display modified tropism. A549-WT, A549-ΔCAR, and HeLa cells were infected by 10 vector particles per cell (vpc) of HAdV-C5-WT (WT) or ADEVO-selected variants. At 3 h post infection (hpi), internalized AdV genomes were quantified. ADEVO-selected variants do not display preferential infection of the A549-ΔCAR cells in which they were selected compared with other cell lines, while HAdV-C5-WT infected A549-ΔCAR cells efficiently. *n* = 6, two independent experiment repeats, error bars: maximum-minimum. (B) A549-ΔCAR cells are permissive to non-modified HAdV-C5 infection. A549-ΔCAR and A549-WT cells were infected at 20 vpc by a luciferase-expressing HAdV-C5 vector. Luciferase luminescence was quantified at 24 hpi, showing relatively strong viral transgene expression in A549-ΔCAR cells. *n* = 2. (C) The SVA variant replicates faster than HAdV-C5-WT. A549-WT cells were infected with 10 vpc of HAdV-C5-WT (WT) or SVA variant. At regular intervals, culture supernatant was collected and excreted VPs were quantified. Alternatively, cells were harvested, washed thoroughly, and intracellular AdV genomes were quantified. *n* = 6, two independent repeats, data points: geometric mean ± standard deviation. SVA and HAdV-C5-WT titers at each time point were normalized on the respective internalized genome titers at 3 hpi and compared by Welch T test (Mann-Whitney *U* tests confirmed the significance in all cases where t test indicated it). ns, *p* > 0.05. ∗∗0.01 > *p* > 0.001. ∗∗∗0.001 > *p*. Two-sided *p* values. (D) The LRT, SRG, and SVA variants lyse efficiently a wide array of cancer cell lines. Cancer cell lines were infected by HAdV-C5-WT or ADEVO-selected variants at various multiplicities of infection ranging from 1 to 300 vpc. Several days later (3 days post infection [dpi] for A549-WT cells, 5 dpi for HCC827 and Hs578T cells, 6 dpi for A549-ΔCAR, HeLa and MiaPaCa2 cells and 7 dpi for EAhy926 cells), live cells were stained with crystal violet. UT, untransduced cells; WT, HAdV-C5-WT. Two to four experiment repeats were performed; representative pictures are shown. (E) CCK8 quantification of infected A549-ΔCAR and A549-WT cells viability. Before crystal violet staining (Figure 3D), the viability of cells infected with 3 vpc or 1 vpc was quantified and normalized on the viability of uninfected cells cultivated in the same conditions. *n* = 3, three independent experiment repeats, error bars: maximum-minimum. (F) Schematic representation of the generated replication-selected vectors carrying the LRT, SRG, or SVA fiber inserts as well as a hTert promoter controlling E1 gene expression. (G) CCK8 quantification of infected A549-ΔCAR and A549-WT cells viability. Before crystal violet staining (Figure 3H), the viability of cells infected with 3 vpc or 1 vpc was quantified and normalized on the viability of uninfected cells cultivated in the same conditions. *n* = 1. hT-WT, HAdV-C5-hTert with WT fiber; hT-LRT/SRG/SVA, HAdV-C5-hTert with fiber insert. (H) The replication-selective variants lyse cancer cells efficiently. A549-WT and A549-ΔCAR cells were infected by replication-selective AdVs at multiplicities of infection ranging from 1 to 300 vpc. Several days later (5 dpi for A549-WT cells, 6 dpi for A549-ΔCAR cells), live cells were stained with crystal violet. Three experiment repeats were performed, representative pictures are shown. (I) Replication-selective variants do not display modified infectivity. A549-WT and A549-ΔCAR cells were infected by 10 vpc of replication-selective AdVs. At 3 hpi, internalized AdV genomes were quantified. *n* = 4, error bars: maximum-minimum.

The purified variants were compared with each other and with WT HAdV-C5 for their infectivity, replication speed, and cancer cell lysis strength in target and non-target cell lines. Contrary to expectations, none of the variants displayed strongly increased internalization in A549-ΔCAR cells compared with HAdV-C5-WT, which was relatively efficient at transducing this target cell line compared with other cell lines (Figure 3A). The internalization rate in A549-ΔCAR cells was proportional to that in A549-WT cells and, except for the FYK variant, correlated with that in unrelated HeLa cells, indicating that the variants were not retargeted to new receptors. We confirmed that HAdV-C5-WT internalization in A549-ΔCAR cells corresponded not only with abortive particle uptake but also productive transduction by studying transgene expression of a luciferase-expressing vector with HAdV-C5-WT capsid, which confirmed that the infectivity of HAdV-C5-WT in A549-ΔCAR cells reaches approximately 20% of the level observed in A549-WT cells (Figure 3B). This indicates that the selective pressure on the cell entry step was lower than expected and suggests that the variants were selected for other features of their life cycle.

We indeed observed increased cell lysis by the selected variants, and in particular the LRT, SVA, and SRG variants, compared with HAdV-C5-WT (Figures 3D and 3E). This effect occurred not only in A549-ΔCAR cells, but also in all other tested cancer cell lines, whether also derived from lung carcinoma (A549-WT, HCC-827) or from other cancer types (cervical cancer for HeLa, pancreatic adenocarcinoma for MiaPaCa-2, triple-negative breast cancer for Hs578T, and endothelial cells for EAhy926). To test whether the increased cell lysis elicited by the selected variants stemmed from an intrinsically higher toxicity or simply from a more efficient replication, we compared the DNA replication speed of SVA, the most lytic variant, with HAdV-C5-WT. In A549-WT cells, the SVA variant replicated substantially faster than HAdV-C5-WT, both in terms of virus genome copies per cells and VP release in the culture supernatant (Figure 3C). Of note, the selection criterion was completion of full replication cycles rather than strictly transduction, which explains why variants hardly more infectious but strongly replicating and lytic were able to outcompete their competitors.

To confirm whether the properties of the selected RC-Ads could be used for oncolytic applications, we generated HAdV-C5 replication-selective vectors whose E1A gene was placed under control of the tumor-specific hTert promoter and whose fiber knob domain displayed the selected LRT, SRG, or SVA inserts (Figure 3F). As expected, replication-selective variants displayed properties similar to their replication-competent counterparts, namely, enhanced cancer cell lysis (Figures 3G and 3H) without significant infectivity increase (Figure 3I).

**Figure 4:**
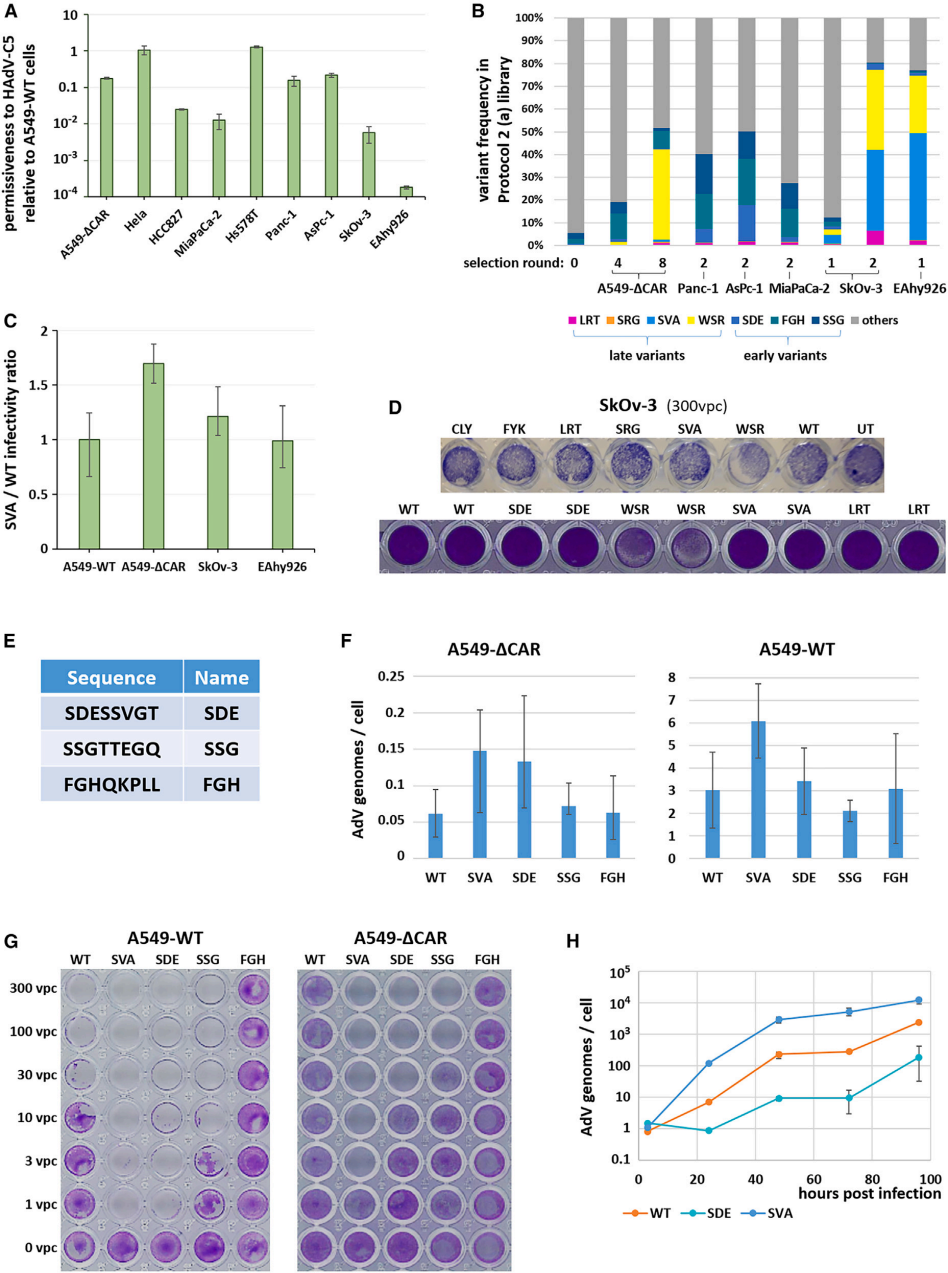
Broad-spectrum lytic variants were systematically selected in a panel of cancer cell lines (A) Cancer cell line permissiveness comparison. Cells were infected at 20 vpc by a luciferase-expressing HAdV-C5 vector. Luciferase luminescence was quantified at 24 h post infection (hpi) and normalized for each cell line on the luminescence levels measured in A549-WT cells as a proxy for permissiveness to HAdV-C5. *n* = 4, error bars: maximum-minimum. (B) Evolution of variant frequencies within protocol 2 (a) library during selection in multiple cancer cell lines. Variants were classified as “early” or “late” based on the selection round at which they achieved their highest frequency in A549-ΔCAR cells (4 and 8, respectively). “others” variants were not dominant or co-dominant in any sequenced library and thereby not represented individually. (C) The late SVA variant is no more infectious than HAdV-C5-WT in low-permissiveness cell lines. A549-WT, A549-ΔCAR, SkOv-3 and EAhy926 cells were infected by 10 vector particles per cell (vpc) of HAdV-C5-WT (WT) or SVA variant. At 3 hpi, internalized AdV genomes were quantified and normalized on the WT average for each cell line. *n* = 3, error bars: maximum-minimum. (D) The WSR variant is the only identified AdV able to lyse SkOv-3 cells. SkOv-3 cells were infected by HAdV-C5-WT or ADEVO-selected variants at 300 vpc (lower multiplicities of infection showed no cell death for any AdV). Eight days later, live cells were stained with crystal violet. UT, untransduced cells; WT, HAdV-C5-WT. (E) Identified early variants. (F) Early variants show no increase in infectivity. A549-WT and A549-ΔCAR cells were infected by 10 vpc of replication-selective AdVs. At 3 hpi, internalized AdV genomes were quantified. *n* = 8, two independent repeats, error bars: maximum-minimum. (G) The SDE and SSG variants, but not FGH, show increased cancer cell lysis. A549-WT and A549-ΔCAR cells were infected by HAdV-C5-WT or ADEVO-selected variants at 1–300 vpc. Four and 6 days later respectively, live cells were stained with crystal violet. WT: HAdV-C5-WT. Two experiment repeats were performed, representative pictures are shown. (H) The SDE variant does not replicate faster than HAdV-C5-WT. A549-WT cells were infected with 10 vpc of HAdV-C5-WT (WT) or SDE or SVA variants. At regular intervals, cells were harvested, washed thoroughly, and intracellular AdV genomes were quantified. *n* = 3, error bars: maximum-minimum.

We repeated the selection of protocol 2 (a) library (the most complex generated library) in other cancer cell lines, namely the pancreatic cancer cell lines AsPc-1, Panc-1, and MiaPaCa-2, as well as the low-permissiveness (Figure 4A) cell lines SkOv-3 (ovarian carcinoma) and EAhy926 (a hybrid of primary umbilical vein endothelial cells and A549 cells). The goal of these selections was to determine whether different variants would be selected than in A549-ΔCAR cells, and especially if variants selected in low-permissiveness cell lines would display improved infectivity rather than accelerated replication and cell lysis. To increase the selective pressure on the infection step, library infection of SkOv-3 and EAhy926 cells was performed in serum-free medium, at a decreased multiplicity of infection and with a shorter infection window before medium change. Library deep sequencing after one or two selection rounds, however, indicated that the selection followed a course similar to selection in A549-ΔCAR cells (Figure 4B): the variants that were most abundant at round 4 (hereafter termed “early variants”) in A549-ΔCAR cells were also among the dominant ones after two selection rounds in pancreatic cancer cells and after one round in SkOv-3, while the so-called late variants became dominant in SkOv-3 cells after two rounds and in EAhy926 cells after only one round. As in more permissive cell lines, the late variants showed enhanced cell lysis in EAhy926 cells (Figure 3D) but (at least in the case of the SVA variant) no substantial increase in infectivity (Figure 4C). However, none of the tested AdVs apart from the WSR variant induced noticeable lysis of SkOv-3 cells (Figure 4D).

Finally, early variants were recloned, purified, and characterized. They did not share the sequence pattern of late variants, with a predominance of non-charged or negatively charged polar amino acids, while FGH had two positively charged amino acids and SDE and SSG none (Figure 4E). The early variants showed no change in infectivity in A549-ΔCAR and A549-WT cells (Figure 4F). FGH was less lytic than the parental HAdV-C5-WT (Figure 4G), contrary to the SDE and SSG variants, although they still seemed to be less lytic than the late variant SVA. Contrary to SVA, the early SDE variant showed no increase in genome replication compared with HAdV-C5-WT (Figure 4H).

## Discussion

Here we describe ADEVO protocols facilitating the generation of highly complex libraries reaching close to 1 million variants (Figure 2A). This represents an approximately hundred-fold improvement compared with the most complex AdV libraries published before the start of this work.^15^ Concomitant with our study, another library generation protocol was published, based on principles comparable to our protocol 1 and achieving similar complexities.^26^ Although ADEVO libraries still fall short of the greatest complexities reported with AAV vector libraries, they are likely to contain improved variants for numerous applications, provided that random modifications are performed in a relevant locus and selection criteria are stringent enough to enrich the improved variants. In addition, we focused here on user-friendly, relatively low-cost protocols requiring only materials readily available in most virology laboratories. Library complexity may be further increased with limited workflow changes, for example, a larger scale library generation or the use of specialized AdV producer cell lines such as HEK293A or AD293 cells. Additional modifications could include the use of semi-random codons to decrease the proportion of non-functional variants with stop codons in the initial libraries.

We used random octopeptides (counting semi-random positions) inserted into the fiber knob HI loop because this type of inserts already facilitated efficient vector retargeting in various rational design studies.^8^^,^^9^^,^^10^ Therefore, the potential library sequence space comprises several billion theoretical peptides, of which less than 0.1% were observed in our libraries (Figure 1C). This means that there may exist more efficient inserts than the ones selected here: although the enriched variants overcame the selective pressure better than the other variants from the libraries, only a small fraction of the total pool of possible variants was generated and submitted to selection to begin with. However, there is to our knowledge no reason to assume that, using the same library generation protocols, library complexity would be higher with inserts of different length. Likewise, limiting insert length to four random amino acids to cover the full sequence space would not have guaranteed that more efficient variants could have been selected. In future applications, our library generation techniques can be used with complementary machine learning algorithms applied to selected sequence patterns in an attempt to virtually sample the billions of variants that were not incorporated into the physical libraries.^27^

Round 0 libraries already showed extensive variations in abundance between variants (Figure 2B), even before selection had begun. This does not seem to stem from bias during variants genome cloning before transfection, given that the most prevalent variants in sequenced genome libraries did not represent more than 0.0008% of these libraries (Table S2). The bias may instead result from random founder effects, whereby limited numbers of transfected AdV genomes find excellent conditions to replicate in producer cells, while others suffer from suboptimal timing, intracellular or intranuclear location, cell health or cell cycle step. Furthermore, differential capsid stability at the VP packaging stage of the AdV life cycle may contribute to abundance discrepancies between variants. It is also possible that certain variants can undergo a second replication cycle before the VP library is harvested. This may explain that inserts with stop codon were already less frequent at round 0 than expected from purely random distribution, ranging from 4% to 12% (Figure 2D) compared with theoretical frequencies of 20% for protocol 1 and 17% for protocols 2 and 3. On the contrary, genome libraries matched the theoretical stop codon frequencies (Table S2), confirming that genome library bias was very limited. Biases in round 0 library composition may be avoided by earlier VP harvest, which may increase library complexity at the cost of lower VP titers. This trade-off may explain the lower complexity of protocols 2(b) and 3 libraries compared with protocols 1 and 2(a) libraries, since the former displayed wider abundance distribution (Figure 2B), fewer inserts with stop codons (Figure 2D), and more extensive producer cell death (data not shown), suggesting that a noticeable proportion of the library variants already amplified through a second generation before round 0 VP library collection.

The lack of highly prevalent variant in genome libraries (Table S2) also shows that the PCR amplification of inserts from library aliquots during NGS sequencing preparation did not cause substantial biases in detected insert prevalence. Likewise, previous studies reported that PCR amplifications before NGS variant calling caused only limited bias in reported library complexity.^28^^,^^29^ These articles recommended to use high input DNA mass, high-performance and high-fidelity polymerases and to maximize the percentage of PCR product sequenced, to decrease the bias in sequencing results. All these guidelines were satisfied by our workflows. Finally, the fact that several of the variants highly enriched after selection had given only a few reads or were not even detected by NGS in round 0 libraries (Figure 2F) indicates that many or most low-prevalence reads correspond to actual variants and not PCR or NGS errors, and that numerous variants were not included in the sequenced round 0 library aliquots. This suggested that library complexity may have been underestimated due to lack of sequencing depth, rather than overestimated through sequencing errors, and warranted the use of statistical modeling to estimate total library complexity. However, modeling seemed to lack robustness. For libraries where more than three library aliquots had been sequenced, we calculated the estimated complexity for all possible combinations of three aliquots and found up to 3-fold variations, depending primarily on the total number of reads per aliquot (Figure S2). Furthermore, the estimates given by different relevant models also varied up to 4-fold. Fortunately, the ratios between the estimated complexities of different libraries were always similar and roughly matched the ratios of observed complexities (i.e., the number of variants identified by NGS in the sequenced aliquots). We therefore assume that statistical models enable a comparison of the relative complexities of different libraries, although some of them may give inaccurate estimates. We ensured estimation consistency by always following the same protocols for NGS and analysis and focused on the model which gave the results closest to the average of all relevant models.

HAdV-C5-WT was able to infect A549-ΔCAR cells relatively efficiently (Figure 3B). Since no trace of residual CAR expression could be detected at the genomic^24^ or at the protein level (data not shown), this permissiveness was likely due to the strong expression by A549-derived cells of secondary AdV receptors including heparan sulfate proteoglycans and integrins.^30^^,^^31^ Therefore, the selective pressure on target cell infectivity was substantially lower than originally desired, which we hypothesized caused the selection of variants with hardly modified infectivity. This could also explain the slow selection process, with no distinct insert sequence pattern having emerged at round 4 (Figure 2E), and extensive variations in variant prevalence still occurring in subsequent rounds (Figure 2F). New selections were thus conducted in nearly non-permissive cell lines, with shorter infection duration, serum-free medium (to avoid fiber-independent infection through VP bridging to cell surface by serum proteins^32^) and lower multiplicity of infection (with the goal of decreasing bulk infections that may occur at high virus doses), to increase the selective pressure on the infection stage. However, variants selected in low-permissiveness cell lines belonged to the already identified late variants. Their selective fitness may be attributed to broad-spectrum improved cell lysis kinetics and replication, which may offset the advantage in infectivity that other variants may have. Therefore, this proof-of-concept study indicates that the selection of retargeted AdVs requires stringent measures such as protocols independent of replication and cell lysis, fully detargeted backbones for library generation that do not bind known AdV secondary receptors, or anti-integrin antibodies or RGD peptides to saturate target cells integrins before library infection.

The most highly enriched variants were re-cloned from assembly backbones after identification and purified individually. This ensures that the differences they displayed compared with HAdV-C5-WT during characterization (Figure 3) come from their fiber insert and not potential mutations elsewhere in the genome that may have arisen during library generation and selection. This was further confirmed by the conservation of variants properties when knob inserts were transferred to the replication-selective backbone. The lytic efficiency of late variants correlated with the electric charge of their insert peptide and with the presence of potential furin cleavage sites. Indeed, the FYK variant has two positively charged amino acids in its insert, CLY has three, WSR, SRG and SVA have five, and LRT six. Meanwhile, the canonical RX(K/R)R furin cleavage site is present once in the SRG and SVA variants inserts and twice in the LRT variant insert, the WSR variant carries one RXXR minimal furin recognition motif, and the ProP software^33^ predicted that the LRT insert indeed had a significant furin cleavage potential (Figure S3). We therefore hypothesize that the potential fiber proteolytic cleavage or the fiber electric charge was responsible for the increased DNA replication and cell lysis. Increases in genome replication and positively charged inserts were nevertheless not shared by the early variants that showed increased lysis (Figures 4E and 4H), suggesting other mechanisms are involved, at least for early variants. Furthermore, the FGH variant persisted at a relatively high frequency in libraries despite lacking increased infectivity and lytic strength, suggesting it relied on artificial cell lysis between each round to spread and/or displays advantages at untested stages of its life cycle such as replication as observed with SVA. The fiber protein was not known to be involved in DNA replication, but the inserts may confer it new functions, for example, the activation or inhibition of new ligands involved in a cellular signaling pathway, respectively, beneficial or detrimental for AdV replication. The inserts may also indirectly influence the replication process for example by modifying the kinetics and efficiency of VP endosomal escape and intracellular trafficking to the nucleus. Investigations of the mechanisms of fiber-mediated replication increase are warranted.

The lower cell lysis ability of the CLY, FYK, and WSR variants compared with LRT, SRG, and SVA observed in most tested cell lines (Figure 3D) could be interpreted as showing a lower fitness of the former variants compared with the latter, since CLY and FYK were selected from protocol 1 library and did not have to compete with the other characterized variants, and WSR seemed to have a slower enrichment rate in A549-ΔCAR cells than its counterparts in protocols 2 and 3 libraries (Figure 2F). However, the WSR variant was the only tested virus able to lyse the largely AdV-resistant SkOv-3 cells (Figure 4D), likely explaining its predominance in the library passaged in this cell line (Figure 4B) and suggesting a different lysis mechanism compared with LRT, SRG and SVA.

All three ADEVO protocols facilitated high complexity library generation. Protocols 2 and 3 libraries performed very similarly, even selecting the same variants with few exceptions. The studied protocol 1 library displayed higher purity than protocols 2 and 3 libraries (Figure 2C) and appeared to reach relative equilibrium faster during selection than library a of protocol 2. Indeed, protocol 1 highly enriched variants could already be identified at selection round 4 (Figure 2F) and the insert logo profile did not substantially change between rounds 4 and 8 (Figure 2E). Moreover, protocol 1 provided more equal round 0 starting titers to all variants before selection (Figure 2B). However, protocol 1 required more time and resources due to the two-step library construction. The variants selected by protocol 1 performed worse than protocols 2 and 3 enriched variants in terms of cell lysis, but this may be due to the insert flanking sequences or the lack of strong selective pressure for the intended goal of tropism retargeting rather than to differences in library generation protocol. The large overlap between variants that were highly enriched in either of the three protocols 2 and 3 libraries indicate that the selection was robust, but also that round 0 libraries already shared numerous variants despite having been generated independently and covering only a limited fraction of the theoretical sequence space. Library complexity may thus have been limited by biases in the composition of random oligonucleotides used for library generation, although these potential biases were not extensive enough as to visibly skew the insert amino acid distribution in the whole library (Figure 2G).

ADEVO libraries may be harnessed for other goals than tropism retargeting, for example escape from neutralizing antibodies and other serum proteins, whose binding to the AdV capsid can cause therapeutic inefficiency or even toxicity.^34^^,^^35^^,^^36^ Detargeting from these undesired ligands could be achieved by random peptide insertion in other fiber knob sites than the HI loops, as well as in other capsid proteins, notably in hexon hyper-variable regions, which are the main binding sites of undesired ligands.^37^^,^^38^ Finally, other types than HAdV-C5 may be used as backbones to combine specific improvements obtained by directed evolution with the natural diversity in potentially clinically interesting AdV features.

In this study, we established user-friendly ADEVO workflows covering random library generation, selection and analysis. We generated highly complexed AdV libraries and selected variants with increased lytic potential. We hope that this study provides the necessary toolkit for a wider use of directed evolution in the generation of novel AdVs for a broad range of applications. Furthermore, we constructed broad-spectrum replication-selective oncolytic vectors with improved lysis *in vitro*. Although OAdV clinical efficiency also depends on parameters so far untested with the new variants, such as the recruitment of immune infiltrates in the tumor, this represents an additional step toward medical applications of ADEVO.

## Materials and methods

### Cell culture

The A549-ΔCAR cell line was previously generated by CRISPR-Cas9 deletion of the CXADR gene expressing the CAR receptor.^24^ A549-WT, A549-ΔCAR, HeLa, HEK293, Hs578T, Panc-1, EAhy926, and MiaPaCa-2 cells were cultivated in DMEM (Pan-Biotech, P04-035911) supplemented with 10% fetal bovine serum (FBS, Pan-Biotech, P40-37500) and penicillin-streptomycin (P/S, Pan-Biotech, P06-07100). HCC827 and AsPc-1 cells were cultivated with RPMI (Pan-Biotech, P04-16500) supplemented with P/S and respectively 20% (HCC827) or 10% (AsPc-1) FBS. SkOv-3 cells were cultivated in McCoy’s 5A Medium (Pan-Biotech, P04-05500) supplemented with P/S and 10% FBS. All cells were kept at 37°C under a humidified atmosphere with 5% CO_2_. Three hours post AdV infection (hpi), cell media were changed and FBS concentration was decreased to 2% (or 5% for HCC827 cells). All cell lines tested negative for mycoplasma infection using the VenorGeM Classic kit (Minerva Biolabs, #11-1025). Serum-free optiMEM medium was obtained from Gibco (#31985070).

### AdV acquisition

PCRs were performed using the Phusion DNA Polymerase (New England Biolabs, #M0530S) following the manufacturer’s instructions. All oligonucleotides used in this study are listed in Table S1.

AdV genome recombineering was performed as described in Zhang et al*.*^39^ Briefly, selection markers were PCR amplified using primers with overhangs to obtain homology arms able to recombine with the sequences flanking on both sides the locus to be modified. Selection markers contained an ampicillin resistance gene for positive selection and the ccdB toxin for negative selection and were inserted into plasmids containing the AdV genome to be modified by homologous recombination in bacteria expressing the λRed recombinase and the mutated ccdA antitoxin (GB05-red with GyrA mutation of Arg462Cys). In a second step, the selection marker was removed and replaced by the desired sequence flanked by homology arms, coming either from an oligonucleotide in the case of point mutations or from PCR amplification when larger sequences needed to be inserted. This homologous recombination was performed in bacteria expressing the RecET recombinase system.^40^

The HAdV-C5-hTert vector was constructed by replacing HAdV-C5-WT nucleotides 445–544 with the human telomerase promoter PCR-amplified from human cell genomic DNA.

Protocol 1 assembly backbone was obtained by replacing all HAdV-C5 sequences between the fiber gene and the right ITR with a PacI restriction site. The deleted sequences plus a 20-nt-long homology arm in 5′ of the fiber gene were subcloned into a pJet plasmid (CloneJET PCR cloning kit, Thermo Fischer Scientific, K1231) and flanked by BstBI restriction sites to obtain the shuttle plasmid. BamHI-HF (New England Biolabs, #R3136S) and SpeI-HF sites (New England Biolabs, #R3133S), separated by two nucleotides as a frameshift to avoid rescue of undesired parental virus during library production, were inserted between the codons 546 and 547 of the fiber gene, corresponding with the HI loop position^15^ (Figure 1B). To this end, the whole shuttle plasmid was PCR amplified with overlapping primers containing the desired insert and recircularized by NEBuilder (New England Biolabs, #E2621L) recombination.

Protocol 2 assembly backbone was obtained by insertion of a SwaI restriction site between the codons 546 and 547 of the fiber gene of HAdV-C5-WT. This introduced a frameshift and stop codon aimed to avoid rescue of undesired parental virus during library production. The same mutation was introduced in HAdV-C5-hTert. Protocol 3 assembly backbone was derived from the protocol 2 assembly backbone by replacing the bacterial plasmid backbone so that the final product does not contain SwaI restriction sites except the one in the fiber gene, and contains the int5 gRNA sequence (tatattattgatgatgCCTC) followed by a CGG PAM at the end of both ITRs, as described in Riedl et al.*,*^21^ to facilitate CRISPR-mediated genome linearization (Figure 1B).

To purify the ADEVO-selected variants, oligonucleotides containing their inserts and surrounding sequences were reassembled into the protocol 2 assembly backbone or the HAdV-C5-hTert-SwaI backbone using the same methods as for random oligonucleotides during protocol 2 library production. The re-cloned ADEVO-selected variants, as well as the HAdV-C5-ΔFiber virus and HAdV-C5-WT and HAdV-C5-hTert controls, were rescued and purified as described in Jager et al.^41^ and titrated by optical density measurements as well as qPCR using a CFX96 Real-Time System machine (BioRad) and the my-Budget 5× EvaGreen qPCR-Mix II (Bio-Budget, #80–5901000) following the manufacturer’s instructions.

The luciferase-expressing HAdV-C5 vector has already been described in Zhang et al*.*^39^

### Cross-packaging assay

Adherent HEK293 cells grown to 80% confluency were transfected using the jetOPTIMUS reagents (Polyplus) following the manufacturer’s instructions with model mini-libraries consisting in SwaI-linearized HAdV-C5-WT and HAdV-C5-ΔFiber genomes at a 1:1 M ratio, with varying total quantities of transfected genomes per cell. Cells and media were harvested when a cytopathic effect (CPE) was observed. The lysates underwent four cycles of freeze and thaw followed by benzonase (Emprove expert benzonase endonuclease, Merck #101695) digestion of non-encapsidated DNA at 37°C for 30 min with gentle shaking using 125 U enzyme per milliliter of lysate. Progeny VPs were titrated by qPCR from lysate dilutions, while subconfluent HEK293 cells were infected using 40% of total lysate volume per well of six-well plates. At 3 hpi, infected cells were washed five times to eliminate non-internalized VPs and DNA was extracted using the NucleoSpin Tissue kit (Macherey-Nagel #740952-250). Internalized AdV genomes were titrated by qPCR using a forward primer in the U exon, a reverse primer within the fiber gene to titrate HAdV-C5-WT genomes, and a reverse primer downstream of the fiber gene (Table S1) to titrate HAdV-C5-ΔFiber genomes. The cross-packaging rate was calculated as the proportion of HAdV-C5-ΔFiber genomes among all internalized AdV genomes.

### Random library production

Shuttle plasmids and protocols 1, 2, and 3 assembly backbones were digested for 24 h using, respectively, the BamHI-HF and SpeI-HF; PacI; PacI then SwaI (24 h each); and SwaI restriction enzymes (New England Biolabs), then purified with phenol-chloroform-isoamyl alcohol (Roth, #A156.3), precipitated with ethanol and resuspended in pure water.

The first phase of protocol 1 consists in constructing a shuttle library following methods already established for random peptide display on AAVs.^20^ NEBuilder homologous recombination was performed using 225 fM of BamHI-HF-SpeI-HF-digested shuttle plasmid and 2.25 pM of single-stranded oligonucleotides containing 28-nt-long homology arms to each end of the BamHI-HF-SpeI-HF-digested shuttle plasmid and a central NNK7 motif coding for a random heptapeptide with lower redundancy (Figure 1C). The recombination products were purified with phenol-chloroform-isoamyl alcohol, precipitated with ethanol, and resuspended in pure water, then electroporated in competent *E. coli*. After 1 h recovery in pure LB media, the electroporated bacteria were cultivated overnight in 100 mL of LB media supplemented with 50 μg/mL ampicillin. The shuttle library was extracted using the ZymoPure II plasmid midiprep kit (Zymo Research, #D4201) and digested with BstBI (New England Biolabs) for 24 h, then purified with phenol-chloroform-isoamyl alcohol, precipitated with ethanol, and resuspended in pure water.

Protocol 1 genome libraries were reassembled by NEBuilder homologous recombination using 375 fM of the BstBI-linearized shuttle library and 375 fM of the PacI-linearized protocol 1 assembly backbone. Protocols 2 (respectively protocol 3) genome library reassembly was performed in one step using 4.5 pM (respectively 2.25 pM) random oligonucleotides and 225 fM PacI-SwaI-digested (respectively SwaI-digested) assembly backbone for three fragments (respectively two fragments) NEBuilder homologous recombination. The recombination products were purified with phenol-chloroform-isoamyl alcohol, precipitated with ethanol and resuspended in pure water.

For all protocols, round 0 libraries were obtained by transfecting 4.8 μg of genome library in 30 million 80% confluent low passage number HEK293 cells (corresponding with approximately 4,000 reassembled genomes per cell if the reassembly was 100% efficient) using the jetOPTIMUS reagents (Polyplus, #101000006) following the manufacturer’s instructions. In the case of protocol 3, *in vivo* AdV genome linearization was facilitated by cotransfection of 9.6 μg pAR-Int5-Cas9-Amp helper plasmid as described in Riedl et al*.*^21^ For all libraries, CPE was observed 3 days after transfection and cells and media were harvested. The lysates were frozen and thawed four times then used for selection.

### AdV selection

At each selection round, to determine library infection titers, target cells cultivated in 24-well plates were transduced using 50, 10, or 2 μL of the lysate obtained from the previous selection round. At 3 hpi, cells were washed five times to eliminate non-internalized VPs and harvested. DNA was extracted using the Macherey-Nagel Nucleospin Tissue mini kit and internalized genomes were titrated by qPCR. Subsequently, 10 million confluent cells were infected by the desired libraries at 0.5 infectious units per cell in culture medium (except for EAhy926 and SkOv-3 cells which received 0.1 infectious units per cell in optiMEM). Media were changed at 3 hpi (except for EAhy926 and SkOv-3 cells which were incubated 1 h at 4°C then 10 min at 37°C before media change), and cells and media were harvested 4 days post infection and frozen and thawed four times. This lysate would then be used for the next selection round. Eight rounds of selection were performed in A549-ΔCAR cells, one in EAhy926 cells and two in the other tested cell lines.

### NGS

Library purity and complexity was assessed by NGS after round 0, round 8 and optionally rounds 1, 2, or 4. Aliquots consisting of 0.25% of the total lysate volume were taken from the desired libraries before freeze and thaw cycles had been applied and were processed for NGS. Cells were pelleted by centrifugation at 6,000×*g* for 1 min. The pellet was resuspended in benzonase buffer (50 mM Tris-HCl pH = 8.5, 2 mM MgCl_2_) and frozen and thawed four times. Both the supernatant and the resuspended pellet were incubated with 1 U/μL benzonase (Emprove expert benzonase endonuclease, Merck #101695) at 37°C for 1 h with gentle shaking to digest non-encapsidated DNA. In our hands, this treatment facilitated the digestion of more than 99.9% of non-encapsidated DNA while leaving encapsidated viral DNA untouched (Figure S4). Benzonase was then inactivated and AdV genomes were released by incubation in TE buffer with 0.5 mg/mL proteinase K, 5 mM EDTA, and 0.5% SDS for 3 h at 56°C with gentle shaking. Proteinase K was heat inactivated at 80°C for 10 min, then the cell and media samples were regrouped and DNA was purified with phenol-chloroform-isoamyl alcohol, precipitated with ethanol, and resuspended in pure water. Variants inserts and surrounding regions were PCR amplified using the entirety of purified DNA, PrimeStar max DNA polymerase (Takara Bio, #R045A) and barcoded primers (Table S1) with a total of 30 cycles. The 174-nt-long (protocol 1) or 171-nt-long (protocols 2 and 3) amplicons were separated from primers and free nucleotides by electrophoresis in a 2% agarose gel then gel extracted using the MyBudget double pure kit (Bio-Budget, #55–3000) and sequenced by Eurofins Genomics under the NGSelect amplicon adaptor ligation package. Up to six library aliquots were pooled together for sequencing and separated during bioinformatic analysis thanks to their different barcodes. Primer barcodes were 4 nt long and had at least three bases different from each other to minimize the risk of misidentification.

Three aliquots of the round 0 libraries were processed and sequenced to enable statistical estimation of library complexity.

NGS output reads were subjected to quality control to exclude library contaminants including insert-less parental genomes, as well as PCR and sequencing errors. Criteria for read exclusion were lack of read pairing within the same library aliquot, incorrect read length, lack of appropriate primer barcode, mutations in the sequences flanking inserts on either side, NGS quality score inferior to 30 on the insert sequence, and mismatch between paired reads. Reads with unexpected sequences at the two nucleotide positions directly preceding the insert or the two nucleotides positions directly following it were considered as having incorrect flanking sequences, since it meant that the insert could not be identified.

WT fiber sequences and parental fiber sequences (corresponding with the assembly backbones) were counted as library contaminants. Inserts that passed all quality controls were translated to obtain their amino acid sequences. Unique insert sequences were numbered and listed alongside their read counts in the corresponding library aliquots.

NGS variant calling data was analyzed on python using the packages pandas,^42^ numpy,^43^ and Bio.Seq.^44^ The package matplotlib.pyplot^45^ was used for graphical representations.

### Infectivity assays

Cells cultivated in 24-well plates were infected by 10 vpc of ADEVO variants or HAdV-C5-WT. At 3 hpi, cells were washed five times to eliminate non-internalized VPs and harvested. DNA was extracted using the Macherey-Nagel Nucleospin Tissue mini kit and internalized genomes were titrated by qPCR.

### Luciferase assays

Cells cultivated in 96-well plates were infected by 20 vpc of luciferase-expressing HAdV-C5 vectors. Media was changed at 3 hpi and luciferase luminescence was measured at 24 hpi using the Nano-Glo Luciferase Assay (Promega, #N1130) kit, a TECAN infinite f plex plate reader and black 96-well luciferase plates (Thermo Fisher Scientific Nunc A/S).

### Replication assays

Cells cultivated in 24-well plates were infected by 10 vpc of the chosen AdVs. Media were changed at 3 hpi. At the designated time points, cells were either washed five times and harvested for titration of intracellular AdV genomes performed as for infectivity assays or media were collected and replaced to titrate excreted progeny VPs. To this aim, 250 μL of media were treated with 125 U/mL benzonase at 37°C for 30 min with gentle shaking to digest non-encapsidated DNA. Benzonase was then inactivated and AdV genomes were released by incubation in TE buffer with 0.5 mg/mL proteinase K, 5 mM EDTA, and 0.5% SDS for 3 h at 56°C with gentle shaking. Proteinase K was heat-inactivated at 80°C for 10 min, then AdV genomes were purified with phenol-chloroform-isoamyl alcohol, precipitated with ethanol, resuspended in pure water, and titrated by qPCR.

### Cell lysis assays

Cells cultivated in 96-well plates were infected by 1, 3, 10, 30, 100, or 300 vpc of ADEVO variants or HAdV-C5-WT and media were changed at 3 hpi. When CPE was observed for at least one variant in the 1 vpc condition, viability measurements were performed for the 1 vpc and 3 vpc conditions of all AdVs and two wells of uninfected cells with the Cell Counting Kit 8 (CCK8, Sigma-Aldrich #96992). Subsequently, all cells were washed twice with PBS, fixed for 15 min at room temperature (RT) in PBS +2% paraformaldehyde, and washed twice with PBS. Live cells were stained for 10 min at RT in methanol +2% crystal violet. Pictures were taken after thorough washing and overnight drying.

### Statistics

Total library complexity was estimated using Anne Chao’s simple capture-recapture Mh-type model^25^ after counting the overlap of variant composition between at three library aliquots. If more than three aliquots had been sequenced for a library, the average of the complexity estimates computed for each possible set of three aliquots was considered. More details on statistical modeling are reported in Figure S2.

## Data and code availability

The main Python codes used in this study to characterize NGS-sequenced libraries are available on zenodo in open access (https://doi.org/10.5281/zenodo.10083525). All other data necessary for the analysis and reproduction of the results presented in the article are available in the article and supplements or will be made available by the authors upon reasonable request.

## Acknowledgments

We are grateful to Zhi Hong Lu (Washington University School of Medicine) for the insightful discussion on potential pathways of fiber inserts gain of function. This work was supported by the 10.13039/501100001659German Research Foundation [DFG EH 192/5-1 to A.E.]; the internal research grant of the Witten/Herdecke University [IFF 2022-22 to E.S.]; and the internal MD/PhD program of the Witten/Herdecke University (to E.S.). Funding for open access charge: 10.13039/501100001659Deutsche Forschungsgemeinschaft [DFG EH 192/5-1 to A.E.].

## Author contributions

E.S.: conceptualization, methodology, software, investigation, writing – original draft, visualization. J.F.: methodology, resources, writing – review and editing. K.S.: methodology, investigation. L.D.: investigation. N.B.: investigation. A.A.: investigation. E.E.-S.: Methodology. W.Z.: resources. A.W.: methodology. M.C.-C.: methodology. L.L.: conceptualization, writing – review and editing. Z.R.: conceptualization, resources, writing – review and editing. A.E.: conceptualization, writing – review and editing, project administration, funding acquisition.

## Declaration of interests

J.F. and Z.R. are co-inventors in patent application EP20198944 by the Albert Ludwig University of Freiburg that describes the use of CRISPR/Cas-mediated rescue of recombinant AdVs from circular plasmids.

## Supplemental information

Document S1. Figures S1–S4 and Tables S1, S2, S4, and S5

Table S3. NGS inserts counter (nucleotide)

Document S2. Article plus supplemental information
