# Supplementary material for: ADEVO: Proof-of-concept of adenovirus-directed EVOlution by random peptide display on the fiber knob
Source: Mol Ther Oncol. 2024 Aug 30;32(4):200867. doi: 10.1016/j.omton.2024.200867 (PMC11439537; doi:10.1016/j.omton.2024.200867)
Supplement: Document S1. Figures S1–S4 and Tables S1, S2, S4, and S5 [file mmc1.pdf]

OMTON, Volume *32*

Supplemental information

ADEVO: Proof-of-concept of adenovirus-directed EVOlution by random peptide display on the fiber knob

Erwan Sallard, Julian Fischer, Katrin Schroeer, Lisa-Marie Dawson, Nissai Beaude, Arsalene Affes, Eric Ehrke-Schulz, Wenli Zhang, Adrian Westhaus, Marti Cabanes-Creus, Leszek Lisowski, Zsolt Ruszics, and Anja Ehrhardt

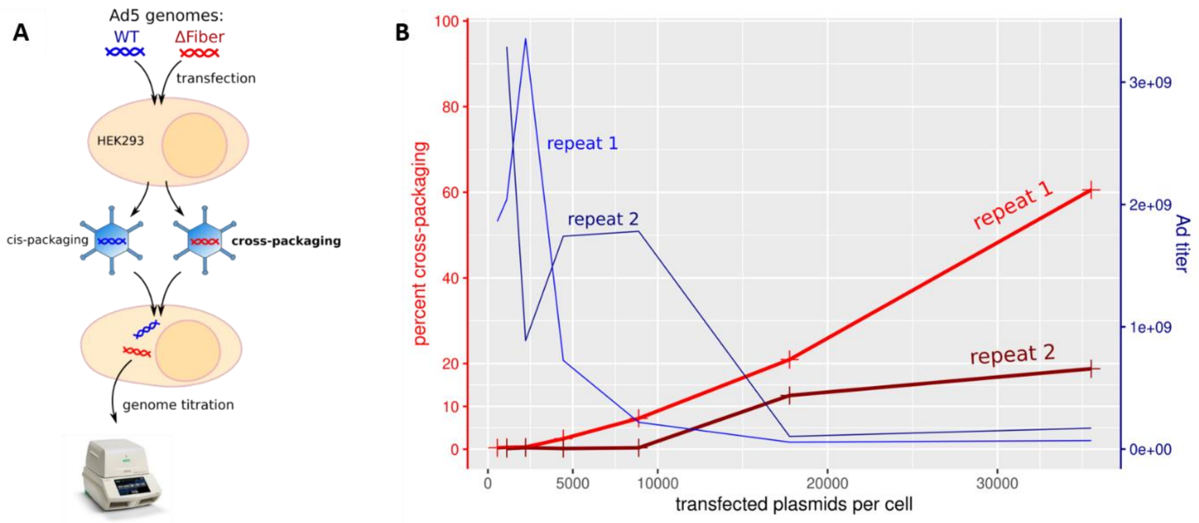

Figure S1. High titer AdV production is achievable under conditions where the cross-packaging rate is low.

A: Experiment design. WT and ΔFiber Ad5 genomes were transfected at a 1:1 ratio. Since only WT genomes can produce functional capsids, the ratio of ΔFiber genomes in cells infected with progeny virions indicates the cross-packaging rate.

B: Progeny titer (blue) and cross-packaging rate (red) as a function of the AdV genome linearized plasmids transfection dose. The experiment was performed twice independently. With our AdV rescue protocols, doses of 5000 transfected plasmids per cell and below facilitated high titer AdV rescue while yielding less than 3% of cross-packaging.

A

Round 0 library	Mth Chao (LB)	Chao 1987 simple model	Chao 1987 complex model	Chao 1992 Mth model	Lee 1994 Mth model	Lee 1994 Mh model
Protocol 1 (#A excluded)	549,984	1,025,166	Not applicable	644,445	837,546	1,711,482
Protocol 1 (#B excluded)	580,218	1,022,536	Not applicable	611,700	817,372	1,708,897
Protocol 1 (#C excluded)	677,169	1,088,894	Not applicable	881,426	807,180	1,768,015
Protocol 1 (#D excluded)	280,719	361,079	Not applicable	236,687	266,712	594,875
Protocol 1 (average ± standard deviation)	522,023 (±147,026)	874,419 (±343,599)		593,564 (±266,548)	682,202 (±277,281)	1,445,817 (±567,950)
Protocol 1 (all 4 aliquots)	747,166					
Protocol 2a (#A excluded)	793,228	1,132,342	Not applicable	472,770	726,972	1,886,984
Protocol 2a (#B excluded)	851,534	1,205,704	Not applicable	503,166	770,010	2,010,719
Protocol 2a (#C excluded)	782,146	1,116,795	Not applicable	465,639	715,729	1,859,827
Protocol 2a (#D excluded)	317,258	398,911	Not applicable	196,188	270,370	654,147
Protocol 2a (average ± standard deviation)	686,042 (±214,543)	963,438 (±378,343)		409,441 (±143,097)	620,770 (±234,768)	1,602,919 (±635,915)
Protocol 2a (all aliquots)	804,727					
Protocol 2b	182,507	245,550	Not applicable	130,109	174,102	409,621
Protocol 3	124,774	166,897	Not applicable	152,019	108,531	259,425

B

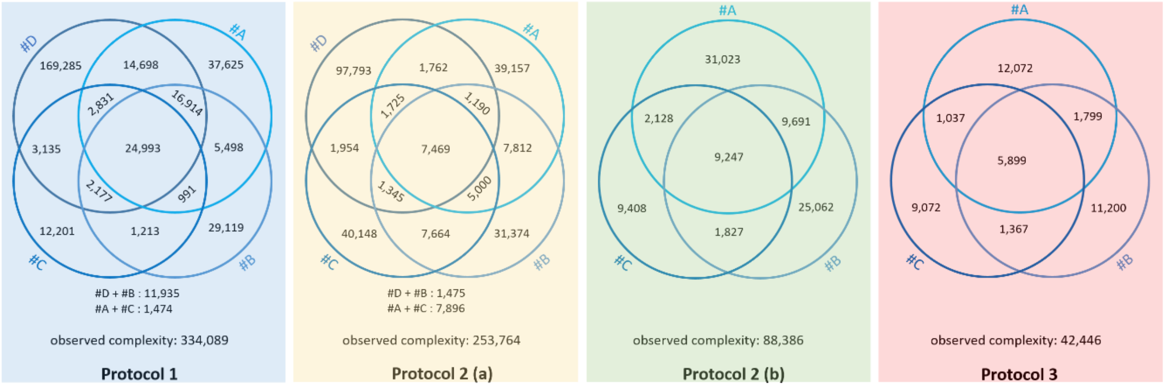

Figure S2. Statistical modeling of library complexity.

A: Complexity estimates yielded by different relevant statistical models, namely Mh and Mth models of Chao (1), Chao et al. (2), Lee et al. (3), or the „Mth Chao (LB) estimate of R's Rcapture package (4), for the different libraries. Complexity estimates inferior to the observed complexity are written in orange. Models did not substantially differ in their relative variability displayed for estimates computed from different combinations of three aliquots from the same library when available. Chao's model was chosen because it came close to the average of all models and never yielded abherrantly low estimates. When four libraries aliquots had been sequenced, the registered complexity was the average of Chao's estimator for all four possible combinations of three aliquots, in order not to introduce bias with libraries for which only three aliquots had been sequenced.

B: Numbers of identified variants and overlap in the three or four sequenced aliquots (#A, #B, #C and/or #D) of round 0 libraries. The observed complexity is the total number of identified variants in all library aliquots combined.

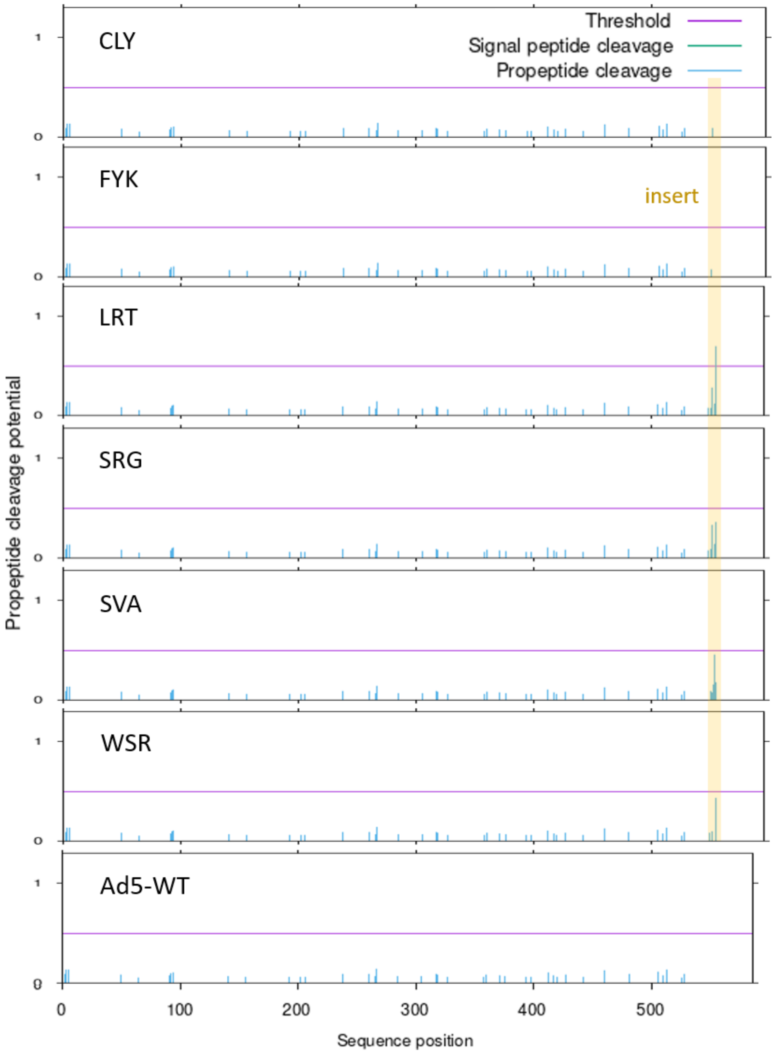

Figure S3. Predicted furin cleavage sites in the selected fiber proteins.

Furin cleavage sites along the whole fiber protein of Ad5-WT or of the selected variants were predicted by the ProP online tool (5). The insert position is indicated in yellow.

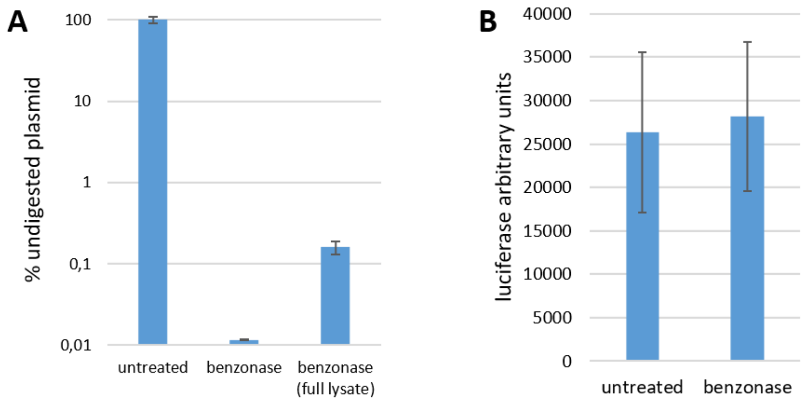

Figure S4. Benzonase treatment eliminates virtually all non-encapsidated DNA without affecting encapsidated DNA.

A: Benzonase digests more than 99.9% of free DNA. Two million HEK293 cells were transfected with 500 billion copies of a plasmid carrying an ampicillin resistance gene. Four hours post transfection, cells and media were harvested, split in three equal groups and processed for benzonase treatment and DNA purification as NGS samples („benzonase" condition), or without benzonase treatment („untreated"), or without separating cells and media but instead conducting four freeze and thaw cycles of full lysate before benzonase treatment („full lysate"). Undigested purified plasmids were numbered by qPCR and normalized on the „untreated" sample. N=2.

B: Benzonase treatment does not disrupt VPs. Two million HEK293 cells were transfected with 1 billion copies of linearized luciferase-expressing HAdV-C5 genomes using the jetOPTIMUS reagents. At 8 dpi, cells and media were harvested and frozen and thawed 4 times. Aliquots of 2% of the total lysate volume were taken and submitted or not to benzonase treatment with the same conditions as for NGS samples preparation except that cells and media were not separated. New HEK293 cells were subsequently infected with the lysate and submitted to luciferase assay in order to quantify infectious VPs whose genomes were left intact. N=2.

Table S1. Primers used in this study.

Blue sequences indicate either one on two random codons (Protocols 1, 2 and 3 random oligonucleotides), or the insert sequence of the primers used for variant recloning. “B” corresponds to C, T or G; “K” to T or G; and “V” to A, C or G.

oligonucleotide	function
cagtttcctcctgttcctgtccatccgcacccactatcttcatgttgttgtttgttca aaaaaaagcccgctc	forward PCR primer, selection marker PCR, Protocol 1 assembly backbone cloning and cloning of the Ad5-ΔFiber virus
tgaattacaacagtactgcgatgagtggcagggcggggcgtaatttaaatg ctgacactctcttaaggtagc	reverse PCR primer, selection marker PCR, Protocol 1 assembly backbone cloning
cagtttcctcctgttcctgtccatccgcacccactatcttcatgttgttgcagatg aagcgcgcaagttaattaaATTTAAATTACGCCCCGCCCTGCCAC TCATCGCAGTACTGTTGTAATTCA	selection marker deletion, Protocol 1 assembly backbone cloning
CTTCGAAcagatgaagcgcgcaag	forward PCR primer, subcloning of the right hand side of the Ad5 genome in a pJet backbone, Protocol 1 shuttle plasmid cloning
GTTCGAAcatcatcaataatataccttattttgg	reverse PCR primer, subcloning of the right hand side of the Ad5 genome in a pJet backbone, Protocol 1 shuttle plasmid cloning
ggACTAGTctGGATCCagttgtgtctcctgtttcctg	forward PCR primer, insertion of BamH1 and Spe1 restriction sites in the fiber gene, Protocol 1 shuttle plasmid cloning
ctGGATCCagACTAGTccaagtgcatactctatgtcattttc	reverse PCR primer, insertion of BamH1 and Spe1 restriction sites in the fiber gene, Protocol 1 shuttle plasmid cloning

taacactaaccattacactaaacggtacacaggaaacaggagacacaacttttgttcaaaaaaaagcccgctc	forward PCR primer, selection marker PCR, Protocol 2 assembly backbone cloning
tagttgtggccagaccagtcccatgaaaatgacatagagtatgcacttggGCTGACACTCTCTTAAGGTAGC	reverse PCR primer, selection marker PCR, Protocol 2 assembly backbone cloning
taacactaaccattacactaaacggtacacaggaaacaggagacacaactATTTAAATccaagtgcatactctatgtcattttcatgggactggtctggccacaacta	selection marker replacement by a Swa1 restriction site, Protocol 2 assembly backbone cloning
ttttctgcaattgaaaaataaacacgttgaaacataacacaaacgattctGCTGACACTCTCTTAAGGTAGC	reverse PCR primer, selection marker PCR, cloning of the Ad5-ΔFiber virus
cagtttcctcctgttcctgtccatccgcacccactatcttcatgttgttgagaatcgtttgtgttatgtttcaacgtgtttatttttcaattgcagaaaa	selection marker deletion, cloning of the Ad5-ΔFiber virus
aggtgttttccgcgttccgggtcaaagttggcgttttattatttgttcaaaaaaagcccgctc	forward PCR primer, selection marker PCR, cloning of the hTert promoter
ctccgtggcagataatatgtctcattttcagtcccggtgtGCTGACACTCTCTTAAGGTAGC	reverse PCR primer, selection marker PCR, cloning of the hTert promoter
aggtgttttccgcgttccgggtcaaagttggcgttttattaCCCTGCGCTGTCGGGGCCA	forward PCR primer, hTert promoter PCR
ctccgtggcagataatatgtctcattttcagtcccggtgtCCCGCTGCCTGAAACTCGCG	reverse PCR primer, hTert promoter PCR
cacaggaaacaggagacacaactGGATCCNNKNNKNNKNNKNNKNNKNNKNCTAGTccaagtgcatactctatgtcatt	Protocol 1 random oligonucleotide
acacaggaaacaggagacacaactATTTBKNNKNNKNNKNNKNNKNNKVNAAATccaagtgcatactctatgtcattt	Protocols 2 and 3 random oligonucleotide
acaggaaacaggagacacaactGGATCCTGTCTGTATAAGGGTTGGCATTCTAGTccaagtgcatactctatgtcat	CLY variant recloning
acaggaaacaggagacacaactGGATCCTTTTATAAGCATTCTGATAATGCTAGTccaagtgcatactctatgtcat	FYK variant recloning
gaaacaggagacacaactATTTTGCGTACGAGGAGGCATAAGCGAAATccaagtgcatactctatg	LRT variant recloning
gaaacaggagacacaactATTTCGCGTGGTAGGAGGCTGAAGAAAAATccaagtgcatactctatg	SRG variant recloning
gaaacaggagacacaactATTTCGGTGGCGCGGAAGCGTCGGAGAAATccaagtgcatactctatg	SVA variant recloning
gaaacaggagacacaactATTTGGTCGCGTTGGCGGAGTATGAGAAATccaagtgcatactctatg	WSR variant recloning

gaaacaggagacacaactATTTCGGATGAGTCGTCGGTGGGTACAAATccaagtgcatactctatg	SDE variant recloning
gaaacaggagacacaactATTTCTTCTGGGACTACTGAGGGTCAAAATccaagtgcatactctatg	SSG variant recloning
gaaacaggagacacaactATTTTTGGTCATCAGAAGCCTTTGCTAAATccaagtgcatactctatg	FGH variant recloning
ggtattgcagcttcctcctgg	forward primer, qPCR titration of all AdVs
accggtttccgtgtcatatgg	reverse primer, qPCR titration of fiber-containing AdVs
acacgttgaaacataacacaaacg	reverse primer, qPCR titration of the Ad5-ΔFiber virus
ggaattgatttgggagagcatc	forward primer, qPCR titration of cellular hB2M gene copies for infectious units titration
caggtcctggctctacaatttactaa	reverse primer, qPCR titration of cellular hB2M gene copies for infectious units titration
AATAgtcagtcaagtttacttaaacgg	forward barcoded primer for NGS, pair 1
AATAggccagaccagtcccatg	reverse barcoded primer for NGS, pair 1
TTATgtcagtcaagtttacttaaacgg	forward barcoded primer for NGS, pair 2
TTATggccagaccagtcccatg	reverse barcoded primer for NGS, pair 2
GGCGgtcagtcaagtttacttaaacgg	forward barcoded primer for NGS, pair 3
GGCGggccagaccagtcccatg	reverse barcoded primer for NGS, pair 3
CCGCgtcagtcaagtttacttaaacgg	forward barcoded primer for NGS, pair 4
CCGCggccagaccagtcccatg	reverse barcoded primer for NGS, pair 4
AGGTgtcagtcaagtttacttaaacgg	forward barcoded primer for NGS, pair 5
AGGTggccagaccagtcccatg	reverse barcoded primer for NGS, pair 5
GTTCgtcagtcaagtttacttaaacgg	forward barcoded primer for NGS, pair 6

GTTCggccagaccagtcccatg	reverse barcoded primer for NGS, pair 6
aagccataccaaacgacgag	forward qPCR primer for AmpR gene, benzonase efficiency test
gtctattaattgttgccgggaag	reverse qPCR primer for AmpR gene, benzonase efficiency test

Table S2. NGS analysis of genome libraries.

Aliquots of one Protocol 1 library and one Protocol 2 libraries were analysed by NGS after genome reassembly and prior to transfection. The dominant variant count is the number of reads corresponding to the most abundant variant of the library.

Genome libraries	Protocol 1	Protocol 2
number of NGS reads that passed quality controls	6,159,383	4,564,775
count of the dominant variant	48	28
percentage of insert reads with stop codons	19.68	17.30
theoretical percentage of inserts with stop codons	19.93	17.35

Table S3: NGS inserts counter (nucleotide)

The following python code takes as input the two .fastq files of paired reads returned by the sequencing company, and a list of parameters to be entered by the user. Reads are allocated to the corresponding library aliquot, undergo quality controls, then true unique inserts are identified. The output is one .csv table per library aliquot, containing the nucleotide sequence of each unique insert with their associated count, in order of decreasing abundance.

Table S4: NGS inserts counter (peptide)
The following python code takes as input an output file of Supplemental code 1, translates all insert nucleotide sequences into amino acid sequences, merges the synonymous inserts and return a new .csv table containing each unique insert peptide sequence with their associated count, in order of decreasing abundance.

```
'''this file takes as input a .csv file of unique NGS inserts and their counts, as
produced by Supplemental Code 1, and gives as output a .csv file of unique corresponding
peptides and counts'''

inputfile='/Example_path_to_the_input_file.csv'
output='/Example_path_to_the_output_file .csv'

from Bio.Seq import Seq
import pandas as pd

rawseq=pd.read_csv(inputfile,sep=',')
peptides=[]
countDNA=[]
errorinsert=0
for k in range(rawseq.shape[0]):
    nt=rawseq.iloc[k,1]
```

```
    if len(nt)%3!=0:
        print('the sequence # '+str(rawseq.iloc[k,0])+' does not have the correct length')
        print(nt)
        errorinsert+=1
    else:
        pep=str(Seq(nt).translate())
        peptides.append(pep)
        countDNA.append(rawseq.iloc[k,2])
    if 'X' in pep:
        print(pep,k,nt)
print('translation finished')
df=pd.DataFrame({})
df['sequence']=peptides
df['count']=countDNA
df=df.sort_values(by=['sequence'])
print('sorting finished')
uniquePeptides=[]
uniqueCounts=[]
memory=''#the last insert at each step of the recurrence, to know if the insert is unique or not
for k in range(df.shape[0]):
    peptide=df.iloc[k,0]
    number=df.iloc[k,1]
    if peptide==memory:
        uniqueCounts[-1]=uniqueCounts[-1]+number
    else:
        memory=peptide
        uniquePeptides.append(peptide)
        uniqueCounts.append(number)
print('unique peptides identified and counted')
print('number of unique peptides: ',len(uniquePeptides))
result=pd.DataFrame({})
result['sequence']=uniquePeptides
result['count']=uniqueCounts
result=result.sort_values(by=['count'], ascending=False)
print('count of the most abundant peptide: ',result.iloc[0,1])
result.to_csv(output)
```

Table S5: Library complexity estimator

The following python code takes as input the peptide sequences and counts tables returned by Supplemental code 2 for the library aliquots considered. It is determined in how many aliquots each insert is present. Then, the number of variants present in exactly 1, 2 or 3 aliquots (in case 3 library aliquots were considered) is counted. Finally, total library complexity is estimated using Anne Chao's simple capture-recapture estimator.

```
import pandas as pd
from random import randint
from statistics import mean,stdev

def WennDiagram(aliquots):
    '''
    aliquots is a list of paths towards peptide or nt inserts counts files from
    the same library. the function identifies in which aliquots each insert was
    identified so that a Wenn Diagram of aliquot overlap can be manually built,
```

```
    and library size can be estimated in the functions below
    '''
    nbaliquots=len(aliquots)
    overlaps=[0 for k in range(2**nbaliquots)]
    # this list indicates the overlaps between aliquots following a basis 2 indexation.
    # for example, if there are 4 aliquots and an insert is present in aliquots
    # 0 and 2 but not 1 and 3 (python numbering so there is an aliquot #0), the
    # insert will be counted at the position 2**0 + 2**2 = 5 (also python numbering)
    insertnotfused=[] # contains the list of insert in each aliquot
    nextinserts=[]
    nbinserts=[]
    index=[]
    stopsign=[]
    for aliquot in aliquots:
        insertlist=list(pd.read_csv(aliquot)['sequence'])
        insertlist.sort()
        nextinserts.append(insertlist[0])
        nbinserts.append(len(insertlist))
        insertlist.append('z')
        # this is the stop mechanism. In the next loop, the end of the list is
        # detected when the studied "insert" is 'z'. All true inserts will come
        # before this one in the alphabetic order
        insertnotfused.append(insertlist)
        index.append(0)
        stopsign.append('z')
    while nextinserts!=stopsign:
        studiedinsert=min(nextinserts)
        score=0
        for k in range(nbaliquots):
            if nextinserts[k]==studiedinsert:
                score+=2**k
                # now let's update nextinserts
                index[k]+=1
                nextinserts[k]=insertnotfused[k][index[k]]
        overlaps[score]+=1
    return overlaps

result=WennDiagram(['/path_to_aliquot_1_inserts_peptide_table.csv','/path_to_aliquot_2_inserts
_peptide_table.csv','/path_to_aliquot_3_inserts_peptide_table.csv.csv'])

def overlaps3toSum(ov):
    '''transforms a list of overlaps for 3 aliquots to a list which counts how
    many inserts were found in respectively 1, 2 or 3 libraries'''
    return [ov[1]+ov[2]+ov[4],ov[3]+ov[5]+ov[6],ov[7]]

# execute the following lines only if the “result” list was built using the WennDiagram function
from exactly three library aliquots
```

```
OverlapsCounts=overlaps3toSum(results)
library_complexity=OverlapsCounts[0]+OverlapsCounts[1]+OverlapsCounts[2]+OverlapsCount
s[0]**2/(2*OverlapsCounts[1])
print(library_complexity)
```

Supplemental references

1. Chao, A. (1987) Estimating the Population Size for Capture-Recapture Data with Unequal Catchability. *Biometrics*, **43**.

2. Chao, A., Lee, S.M. and Jeng, S.L. (1992) Estimating Population Size for Capture-Recapture Data When Capture Probabilities Vary by Time and Individual Animal. *Biometrics*, **48**.

3. Lee, S.-M. and Chao, A. (1994) Estimating Population Size Via Sample Coverage for Closed Capture-Recapture Models. *Biometrics*, **50**.

4. Baillargeon, S. and Rivest, L.-P. (2007) TheRcapturePackage: Loglinear Models for Capture-Recapture inR. *Journal of Statistical Software*, **19**.

5. Duckert, P., Brunak, S. and Blom, N. (2004) Prediction of proprotein convertase cleavage sites. *Protein Eng Des Sel*, **17**, 107-112.
